# Pulmonary Function and Survival 1 Year After Dupilumab Treatment of Acute Moderate to Severe Coronavirus Disease 2019: A Follow-up Study From a Phase 2a Trial

**DOI:** 10.1093/ofid/ofad630

**Published:** 2024-01-03

**Authors:** Jennifer Hendrick, Jennie Z Ma, Heather M Haughey, Rachael Coleman, Uma Nayak, Alexandra Kadl, Jeffrey M Sturek, Patrick Jackson, Mary K Young, Judith E Allen, William A Petri

**Affiliations:** Division of Infectious Diseases and International Health, Department of Medicine, University of Virginia Health System, Charlottesville, Virginia, USA; Department of Public Health Sciences, University of Virginia School of Medicine, Charlottesville, Virginia, USA; Division of Pulmonary and Critical Care Medicine, Department of Medicine, University of Virginia Health System, Charlottesville, Virginia, USA; Division of Infectious Diseases and International Health, Department of Medicine, University of Virginia Health System, Charlottesville, Virginia, USA; Center for Public Health Genomics and Department of Public Health Sciences, University of Virginia School of Medicine, Charlottesville, Virginia, USA; Division of Pulmonary and Critical Care Medicine, Department of Medicine, University of Virginia Health System, Charlottesville, Virginia, USA; Department of Pharmacology, University of Virginia School of Medicine, Charlottesville, Virginia, USA; Division of Pulmonary and Critical Care Medicine, Department of Medicine, University of Virginia Health System, Charlottesville, Virginia, USA; Division of Infectious Diseases and International Health, Department of Medicine, University of Virginia Health System, Charlottesville, Virginia, USA; Division of Infectious Diseases and International Health, Department of Medicine, University of Virginia Health System, Charlottesville, Virginia, USA; Lydia Becker Institute of Immunology and Inflammation, School of Biological Sciences, University of Manchester, Manchester Academic Health Sciences Centre, Manchester, United Kingdom; Division of Infectious Diseases and International Health, Department of Medicine, University of Virginia Health System, Charlottesville, Virginia, USA; Department of Microbiology, Immunology and Cancer Biology, University of Virginia School of Medicine, Charlottesville, Virginia, USA; Department of Pathology, University of Virginia Health System, Charlottesville, Virginia, USA

**Keywords:** COVID-19, dupilumab, post COVID conditions, pulmonary function, type 2 immunity

## Abstract

**Background:**

We previously conducted a phase 2a randomized placebo-controlled trial of 40 subjects to assess the efficacy and safety of dupilumab use in people hospitalized with coronavirus disease 2019 (COVID-19) (NCT04920916). Based on our preclinical data suggesting that downstream pulmonary dysfunction with COVID-19 induced type 2 inflammation, we contacted patients from our phase 2a study at 1 year for assessment of post-COVID-19 conditions.

**Methods:**

Subjects at 1 year after treatment underwent pulmonary function tests, high-resolution computed tomographic imaging, symptom questionnaires, neurocognitive assessments, and serum immune biomarker analysis, with subject survival also monitored. The primary outcome was the proportion of abnormal diffusion capacity for carbon monoxide (DLCO) or 6-minute walk test (6MWT) at the 1-year visit.

**Results:**

Of those survivors who consented to 1-year visits (n = 16), subjects who had originally received dupilumab were less likely than those who received placebo to have an abnormal DLCO or 6MWT (Fisher exact *P* = .011; adjusted *P* = .058). As a secondary endpoint, we saw that 16% of subjects in the dupilumab group died by 1 year compared to 38% in the placebo group, though this was not statistically significant (log-rank *P* = .12). We did not find significant differences in neurocognitive testing, symptoms, or chest computed tomography between treatment groups but observed a larger reduction in eotaxin levels in those who received dupilumab.

**Conclusions:**

In this observational study, subjects who received dupilumab during acute COVID-19 hospitalization were less likely to have a reduced DLCO or 6MWT, with a nonsignificant trend toward reduced mortality at 1 year compared to placebo.

## Teaser

In this follow-up study, subjects who had received dupilumab during their acute COVID-19 hospitalization through a prior phase 2a randomized placebo-controlled trial were less likely to have reduced lung diffusion or abnormal 6-minute walk testing at 1 year postenrollment.

As coronavirus disease 2019 (COVID-19) persists in the United States, it continues to lead to hospitalizations and death due to the increased transmissibility and immune escape characteristics of emerging severe acute respiratory syndrome coronavirus 2 (SARS-CoV-2) variants [1]. Post-COVID-19 conditions (PCCs), which have led to substantial morbidity and mortality, have been estimated to occur in at least 10% of patients with a history of SARS-CoV-2 infection. The percentages rises to >50% for those who required hospitalization [2]. Furthermore, repeated infection with SARS-CoV-2 has been associated with increased death, hospitalization, and PCCs regardless of vaccination status [3]. Although there are currently no recommended treatment options for PCCs, there is evidence that intervention during acute disease can prevent PCCs [4]. Further exploration into the immunopathogenesis of this entity is needed to aid the development of additional management strategies, particularly as current acute COVID-19 therapies against both the virus and with intention for immunomodulation have demonstrated variable and/or modest benefit [5–8], with little known yet about their influence on PCCs.

We have discovered that high plasma interleukin (IL)–13 is associated with COVID-19–induced respiratory failure [9], a finding further validated in other COVID-19 observational studies [10, 11]. IL-13, which signals through the receptor IL-4Rα along with the closely related cytokine IL-4, is involved in eosinophilic inflammation, mucous secretion, goblet cell metaplasia, and fibrosis, and has been regularly implicated in atopic disease [12]. We further found that neutralization of IL-13 in K18-hACE2 C57Bl/6J mice protected the animals from severe infection with SARS-CoV-2, as evidenced by reduced clinical score, weight loss, and mortality [9].

RNA-seq analysis of whole lung tissue taken from infected mice who underwent IL-13 neutralization revealed the most downregulated gene to be *Has1* [9]. This gene encodes a synthase responsible for the synthesis of hyaluronan (HA), a major polysaccharide component of the extracellular matrix that has been implicated in viral-mediated inflammatory pulmonary diseases, including acute COVID-19 [13, 14]. This was mechanistically further supported by an increase in HA deposition in human and mouse lung with SARS-CoV-2 infection [9]. As HA has been demonstrated as a mediator of multiple pulmonary diseases, including pulmonary fibrosis and idiopathic pulmonary hypertension [15], this led to the hypothesis that IL-13, acting as a regulator of HA matrix formation, may be involved in pulmonary dysfunction after recovery from COVID-19 [16].

Based on these data and in an effort to target patients most likely in the host-mediated inflammatory stage of COVID-19 illness, we subsequently conducted a phase 2a randomized, double-blind, placebo-controlled trial (RCT) in those hospitalized with COVID-19 to assess the safety and efficacy of dupilumab, a monoclonal antibody that blocks IL-4Rα, in mitigating respiratory failure and death when added to standard-of-care regimens [17]. Forty subjects were followed prospectively for 60 days. Though the primary endpoint of 28-day ventilator-free survival was not reached, we found that subjects randomized to dupilumab had improved 60-day survival as a secondary outcome: 17 of 19 subjects in the dupilumab group (89%) were alive at day 60 compared to 16 of 21 in the placebo group (76%) [17].

Building on our hypothesis that COVID-19–induced type 2 immune activation leads to a downstream destructive pulmonary process, we contacted subjects previously enrolled in our phase 2a trial 1 year after their original enrollment, inviting them to participate in a follow-up study focusing on assessment of PCCs, as determined by symptoms, neurocognition, pulmonary imaging, immune biomarkers, and pulmonary function.

## METHODS

### Design

Enrollment in the original phase 2a trial occurred from June 2021 until November 2021 (NCT04920916). Subjects were subsequently contacted to return for follow-up visits at 1 year after original enrollment, thus were reenrolled from August 2022 until February 2023. During the visit, patients underwent pulmonary function tests (PFTs), high-resolution computed tomography (HRCT), neurocognitive questionnaires, symptom screening, and blood sample collection for biomarker analysis to determine preplanned study outcomes as listed in Supplementary Table 1. This study was approved by the University of Virginia Institutional Review Board in June 2022.

### Data Collection and Outcomes

Survival data of participants from the original phase 2a RCT were collected via telephone calls and/or via review of the electronic medical record (2 dupilumab subjects, 1 placebo subject). PFTs were obtained at the University of Virginia Pulmonary Function Testing Laboratory under the supervision of qualified technicians per American Thoracic Society guidelines [18]. Testing consisted of spirometry (focusing on forced expiratory volume [FEV_1_] and forced vital capacity [FVC]), 6-minute walk test (6MWT), and measurement of diffusion capacity for carbon monoxide (DLCO). All raw PFT data were compared to population-based Global Lung Function Initiative (GLI) predicted values to determine the percentage of predicted value based on age, sex, height, and ethnicity. A PFT measurement at the 5th percentile or lower was considered abnormal [19].

Neurocognitive testing–generated scores were either converted into a T score to allow for comparison to population average and interpreted per instructions in each user manual or interpreted as stated on the questionnaire itself [20–26]. Within the 3 categories (mood, cognition, and functional status/quality of life; Supplementary Table 2), scores were defined as abnormal if at least 1 of the tests within the category was deemed a variation from population norm per scoring instructions. Symptom screening included questions for assessment of PCCs as listed by the Centers for Disease Control and Prevention [27].

The HRCT chest scans were scored independently by 2 blinded pulmonologists with final scores determined by the average of the 2. There was negligible interrater variability in scoring that would require a third reviewer. Scores were allotted for visualization of subpleural blebs (score of 0 or 2), bronchiectasis (score of 0 or 2), reticular infiltrates (score from 0–4 depending on extent of parenchymal involvement), ground glass opacities (score from 0–4 depending on extent of parenchymal involvement), and honeycombing (score of 0 or 5) within upper, middle, and lower lung zones. Final scores were determined by the sum of scores from each zone.

Serum samples were collected from each subject at 1 year for biomarker analysis using a 47- plex cytokine panel (MILLIPLEX SARS-CoV-2 MAP Human Cytokine/Chemokine/Growth Factor Panel). Serum samples collected at day 0 from the phase 2a study were reanalyzed using this panel to eliminate batch effects.

### Statistical Analysis

Acknowledging that the benefits of randomization from the original RCT had been lost given the nature of this follow-up study, we conducted exploratory analyses for the purpose of hypothesis generation. Data were initially analyzed with the χ^2^ or Fisher exact tests for categorical measures (primary outcome, patient characteristics, symptom screening, and neurocognitive testing) and 2-sample *t* test or Wilcoxon rank-sum test for continuous measures (computed tomography [CT] scores and serum cytokine, chemokines, and growth factor level differences) to assess for potential differences between groups. Mortality (secondary endpoint) was analyzed as a time-to-event outcome using Kaplan-Meier survival and Cox regression. Sex was included in this Cox regression due to the previously determined treatment group imbalance from the original RCT [17] and the completion of the survival dataset. For all other outcomes, the analyses of the treatment effects were adjusted for pertinent variables via either logistic regression or linear regression while being mindful of the reduced cohort sample size. All data analyses were performed using SAS version 9.4 software (SAS Institute).

Analyses of each cytokine, chemokine, and growth factor changes between groups were conducted utilizing nonparametric Wilcoxon test for their skewed distributions. Per our study protocol, we did not plan to make adjustment for multiple comparisons due to the overall small sample size, large number of variables, and exploratory nature of the study. That said, for transparency and as an exploratory analysis, multiple comparison adjustment was conducted in our biomarker analysis using the false discovery rate (FDR).

The primary efficacy endpoint was the proportion of subjects with abnormal PFTs 1 year after enrollment in the phase 2a study. Abnormal PFTs were defined as either an abnormal DLCO, after adjustment for hemoglobin level; compared GLI predicted values based on age, sex, height, and ethnicity; or an abnormal 6MWT defined as a 3% or more decline in blood oxygen during walking. These PFT components were chosen based on prior acute respiratory distress syndrome and COVID-19 data [28–30], which identified them as the most common postrecovery PFT abnormalities in these patient populations. Exertional desaturation was chosen as the 6MWT measure rather than percentage predicted distance based on its known predictive ability for mortality in other pulmonary diseases [31] and to ensure the measure of an oxygenation abnormality regardless of the ambulatory limitations of the participants. Both DLCO and 6MWT measures were chosen as a composite endpoint to broaden our capture and summarization of oxygen diffusion abnormalities in the study population.

As a post hoc analysis, we created a composite endpoint of either abnormal PFTs at 1 year postenrollment or death during the first year. This outcome was analyzed as a binary endpoint in logistic regression rather than a time-to-event outcome. The decision was made due to the intersubject variability in follow-up times between those who completed 1-year follow-up visits and those who died over the first year. Considering this, time from enrollment to PFT measurement was further included in a logistic regression model to account for potential influence of the imbalanced follow-up time. Furthermore, we stratified the post hoc analysis by patient characteristics heuristically (with respect to demographics, comorbidities, concomitant COVID-19 therapeutics received, vaccination status, dichotomized days from symptom onset, and dichotomized day 0 labwork levels, including cell counts, liver function testing, renal function, and inflammatory markers) to identify the subgroups in the population most likely to benefit from dupilumab.

## RESULTS

Survival data were ascertained through follow-up phone calls and medical records for all subjects. Of the 40 originally enrolled patients, there was a total of 11 deaths in the first year (8 in the placebo group, 3 in the dupilumab group). Of the remaining 29 survivors at 1 year, 16 patients consented to reenrollment for follow-up visits (Supplementary Figure 1). There was no difference in demographics, comorbidities, vaccination status, clinical characteristics, or medications received during COVID-19 admission between those subjects who consented versus those who declined follow-up (Supplementary Table 3). Among those 16 subjects who consented to 1-year follow-up (6 in placebo and 10 in dupilumab), there was no difference in these characteristics between the 2 treatment groups (Table 1).

**Table 1.** Demographics, Clinical Characteristics, Comorbidities, and Medications Received During Coronavirus Disease 2019 Admission of Patients Who Followed up at 1 Year Postenrollment

Characteristic	Placebo (n = 6)	Dupilumab (n = 10)
Age, y, median (IQR)	57 (39–75)	56 (48–65)
Male sex	4 (67)	4 (40)
BMI, kg/m^2^, median (IQR)	31 (26–36)	34 (25–43)
Ethnicity, Hispanic	1 (10)	1 (17)
Race		
White	4 (67)	8 (80)
Black	2 (33)	1 (10)
Other	0 (0)	1 (10)
History of cardiac disease	2 (33)	1 (10)
History of kidney disease	1 (17)	1 (10)
History of pulmonary disease	3 (50)	5 (50)
History of neurocognitive dysfunction or stroke	1 (17)	0 (0)
History of smoking	3 (50)	2 (20)
Days till follow-up, median (IQR)	412 (390–434)	426 (394–458)
Vaccinated	3 (50)	5 (50)
N protein level, ng/mL, median (IQR)	419 (–81–919)	543 (–1,717–2,803)
Days from symptom onset to study drug received, median (IQR)	8 (5)	9 (4)
Days in hospital, median (IQR)	5 (1–9)	5 (4–7)
Required supplemental oxygen	6 (100)	9 (90)
Required intensive care unit	3 (50)	1 (10)
Required mechanical ventilation	0 (0)	0 (0)
Received monoclonal antibody	2 (33)	2 (20)
Received steroids	6 (100)	10 (100)
Received remdesivir	5 (83)	8 (80)
Received baricitinib	2 (33)	0 (0)

Data are presented as No. (%) unless otherwise indicated.

Abbreviations: BMI, body mass index; IQR, interquartile range.

**Figure 1.**
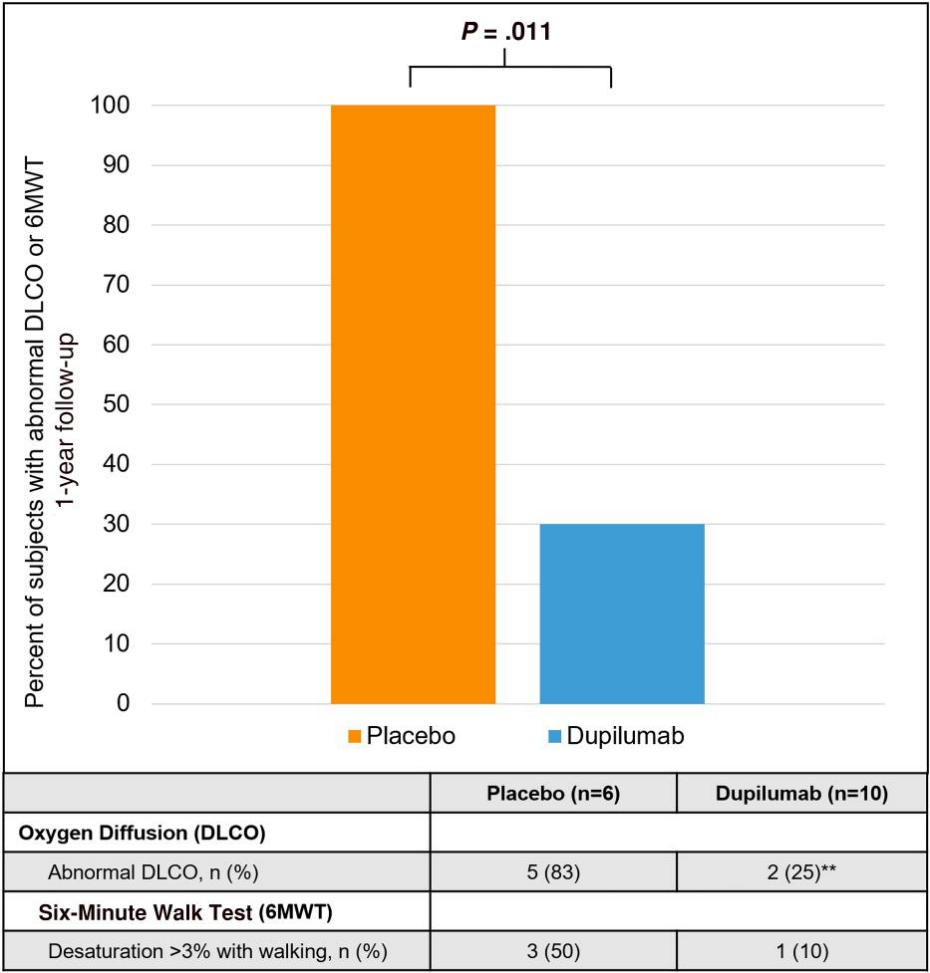
Bar graph illustrating the percentage of patients who had abnormal pulmonary function testing (defined by abnormal diffusion capacity for carbon monoxide [DLCO] or 6-minute walk test [6MWT]) by treatment group at 1-year follow-up. The orange box depicts the patients randomized to placebo during initial coronavirus disease 2019 (COVID-19) admission and the blue box depicts the subjects randomized to dupilumab during initial COVID-19 admission. Actual number and percentages are displayed in the table at the bottom of the chart. **Two missing values.

**Figure 2.**
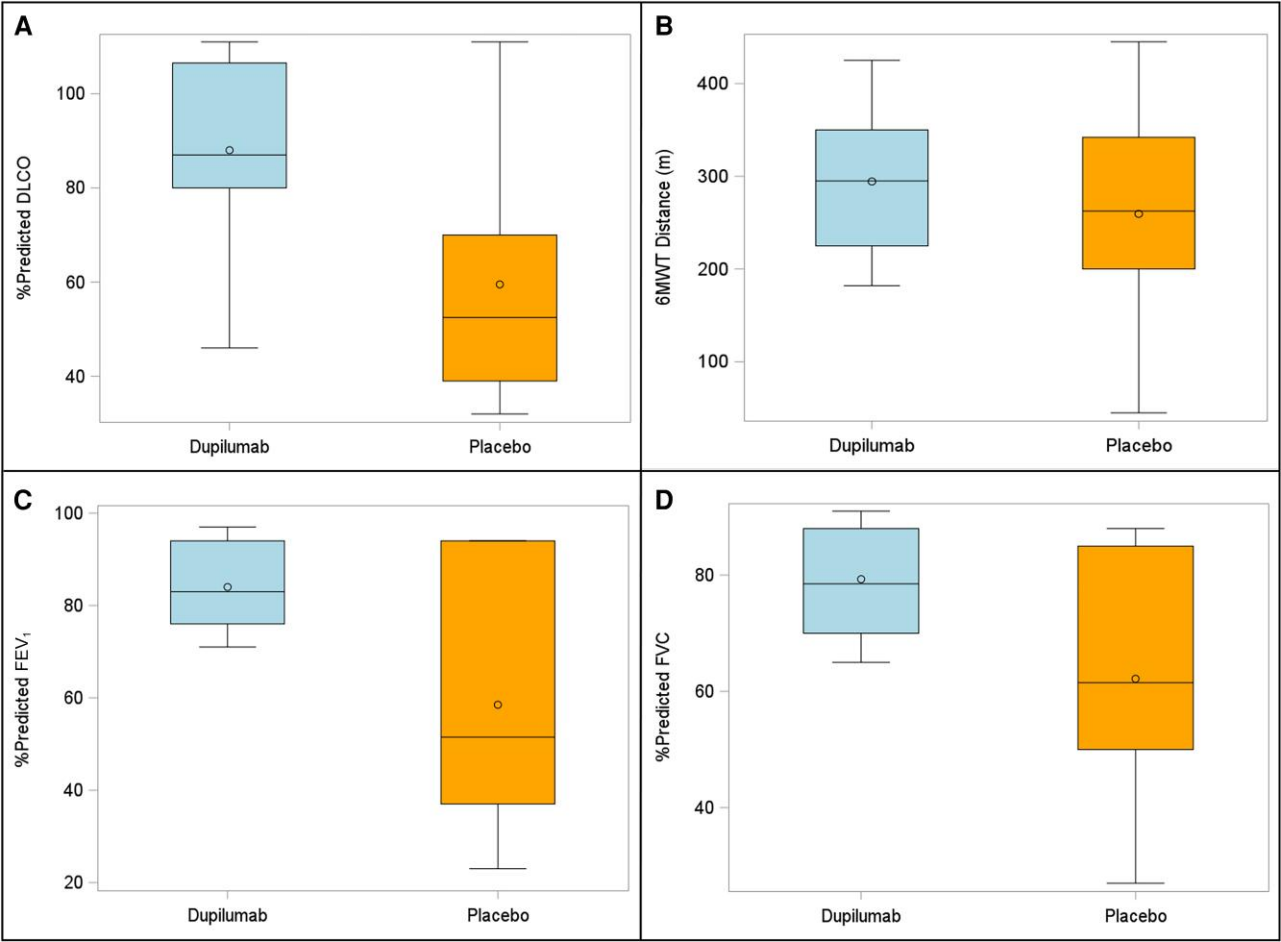
Box plots of percentage predicted pulmonary function measures and 6-minute walk test distance (in meters) between treatment groups. The orange boxes depict the patients randomized to placebo and the blue boxes depict the subjects randomized to dupilumab during initial coronavirus disease 2019 admission. The solid horizontal line within the box is representative of the median value and the open circle within the box is representative of the mean value. *A*, Diffusing capacity of the lungs for carbon monoxide (DLCO). *B*, Six-minute walk test (6MWT). *C*, Forced expiratory volume in 1 second (FEV_1_). *D*, Forced vital capacity (FVC).

We found that all patients in the placebo group (n = 6) had a reduced DLCO or desaturation with 6MWT at 1-year follow-up compared to only 3 of 10 patients in the dupilumab group (Fisher exact *P* = .011, and *P* = .058 from logistic regression after adjusting for time to follow-up, preexisting heart/lung disease, and smoking history; Figure 1). Though not reaching statistical significance, a trend was observed toward reduced percentage predicted FEV_1_, FVC, and DLCO in the placebo group compared to the dupilumab group (Wilcoxon *P* = .12, *P* = .07, and *P* = .08 for FEV_1_, FVC, and DLCO, respectively; see Figure 2 and Supplementary Table 4).

**Figure 3.**
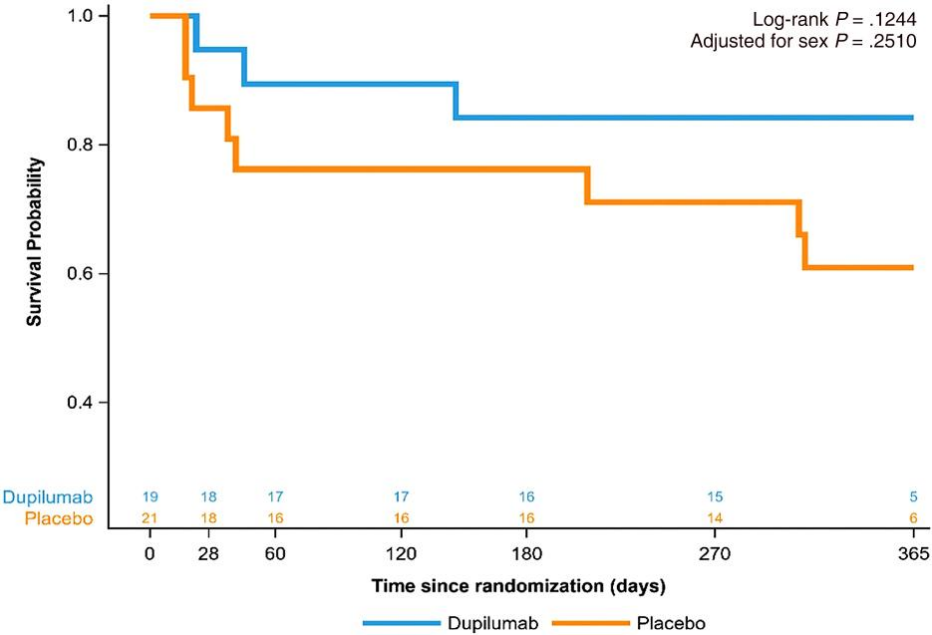
Kaplan-Meier curve depicting 1-year mortality between the 2 treatment groups. Dupilumab group is represented by the blue line. Placebo group is represented by the orange line. Adjusted *P* value indicative of adjustment for sex in the Cox regression.

Of the initial 19 subjects randomized to the dupilumab group, 2 died within the first 60 days, with 1 additional death occurring thereafter within the 1-year period. In contrast, among the 21 placebo subjects, 5 died in the first 60 days, with an additional 3 deaths by 1 year (mortality: 16% in dupilumab vs 38% in placebo, log-rank *P* = .12, *P* = .25 after adjusting for sex in Cox regression; Figure 3). Of the additional 4 deaths that occurred after the original phase 2a study period (60 days), 3 were attributed to respiratory failure (2 occurring during additional hospitalizations and 1 occurring at home), and 1 to septic shock with associated respiratory failure occurring during an additional hospitalization. Two participants in the dupilumab group were unable to complete testing maneuvers required for adequate DLCO measurement.

There was no significant difference in proportion of abnormal mood, cognition, or functional capacity as determined by neurocognitive testing at 1-year follow-up between the 2 treatment groups (Supplementary Table 5). These differences between the treatment groups remain statistically insignificant after adjusting for preexisting neurocognitive dysfunction (for analysis of cognition and functional capacity) or preexisting mood disorder (for analysis of mood variable), age (for analysis of cognition and functional capacity variable), and time until follow-up appointment (for analysis of all 3 variables). Additionally, there was no difference between treatment groups regarding constitutional, mood, respiratory/cardiac, allergic, or sensory symptoms at 1-year follow-up (Supplementary Figure 2), which remained without difference when adjusted for pertinent preexisting conditions and time to follow-up. HRCT scan scores determined that there was no difference in parenchymal abnormalities between the 2 treatment groups (Wilcoxon *P* = .98, adjusted *P* = .96 for preexisting pulmonary disease, smoking status, and follow-up time via linear regression; Supplementary Figure 3).

**Figure 4.**
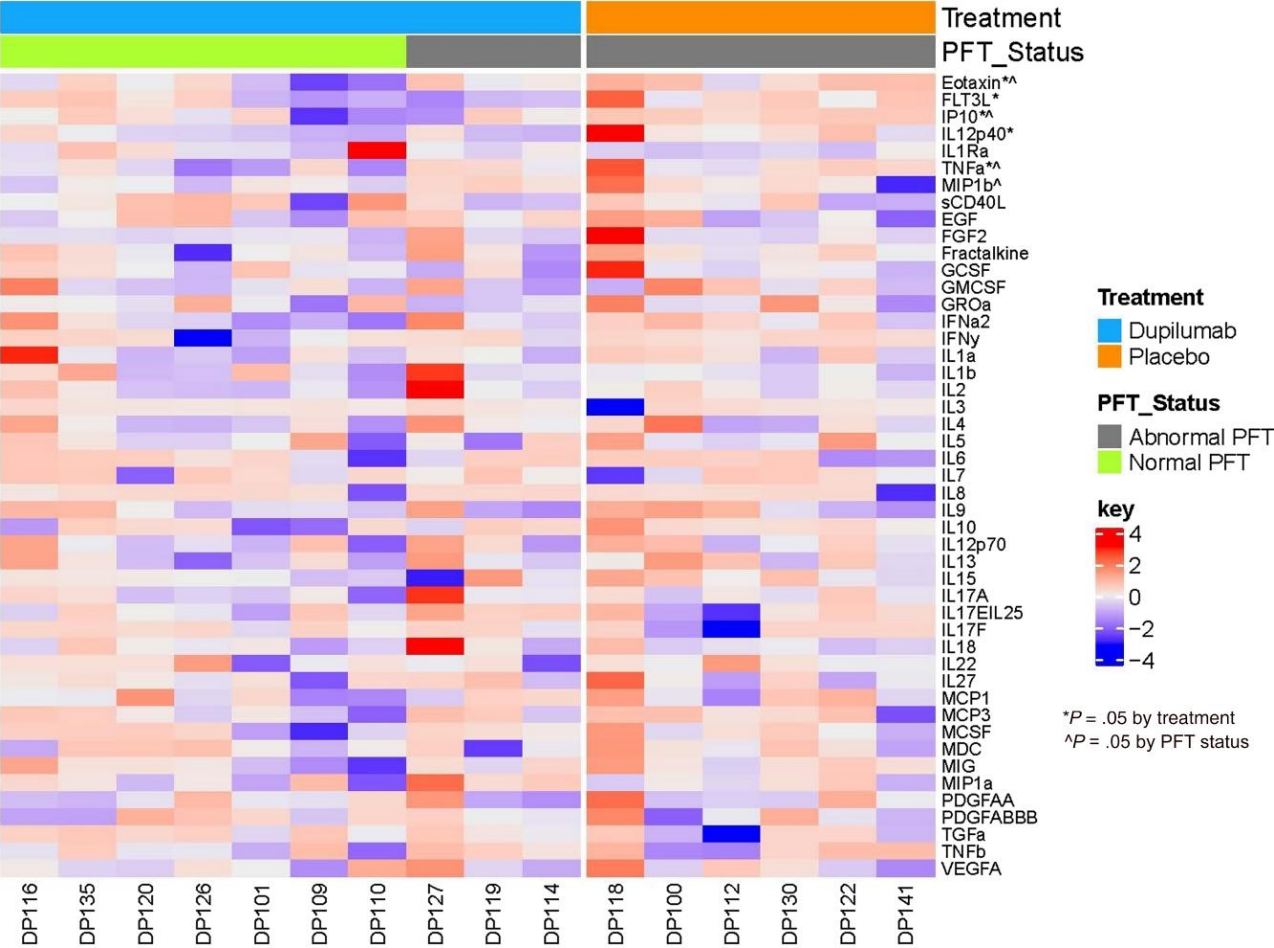
Heatmap showing the standardized differences between baseline and 1-year cytokine, chemokine, or growth factor levels between treatment groups. Each column represents an individual subject labeled by a unique identifier (DP ***). Treatment group is indicated by the top horizontal bar with the blue bar representing the dupilumab group and the orange bar representing the placebo group. Pulmonary function test (PFT) status is represented by the second horizontal bar with subjects who had abnormal PFTs at 1 year (defined as abnormal diffusion capacity for carbon monoxide or 6-minute walk test) represented by the gray bar and those who had normal testing represented by the green bar). Each biomarker is listed on the right of the plot. *Significant differences in 1-year changes between treatment groups. ^Significant differences between 1-year PFT status. *P* = .065 for IL1Ra.

Multiplex analysis of serum cytokines, chemokines, and growth factors revealed that those who had normal PFTs at follow-up had a larger reduction in eotaxin, tumor necrosis factor alpha (TNF-α), and interferon-γ inducible protein 10 (IP-10) over the year period compared to those with abnormal PFTs. Those with abnormal PFTs also had a larger increase in macrophage inflammatory protein–1β at 1-year follow-up. Subjects who received dupilumab had a larger decline in eotaxin, IL-12p40, IP-10, and FMS-like tyrosine kinase 3 (FLT3) and a larger increase in IL-1 receptor antagonist (IL-1Ra) over the 1-year period compared to those who received placebo (Figure 4, Supplementary Table 6). As an exploratory analysis, after the FDR adjustment, none of cytokine, chemokine, and growth factor analyses remained significantly different between the treatment groups (results not shown; all FDR-adjusted *P* > .39).

As part of our exploratory analyses, we stratified our mortality assessment heuristically by multiple patient characteristics, and discovered that those who had lymphopenia (an established indicator of COVID-19 severity [32], defined as a lymphocyte count <1000 cells/µL) at original enrollment appeared to respond better to dupilumab compared to placebo in reducing 1-year mortality (log-rank *P* = .019, and *P* = .077 after adjustment for sex via Cox regression; Supplementary Figure 4). Furthermore, as an ad hoc analysis, for the composite outcome of death or abnormal PFT (DLCO or 6MWT), we found that those who received dupilumab were 77% less likely to die or have an abnormal PFT at 1-year follow-up compared to those who received placebo (odds ratio [OR], 0.23 [95% confidence interval, .06–.87], *P* = .03; and adjusted OR, 0.22, *P* = .06 after adjusting for age, sex, and preexisting pulmonary disease via logistic regression; Supplementary Figure 5).

## DISCUSSION

In this pilot study we found that individuals who received IL-13 and IL-4 signaling blockade during their hospitalization for acute COVID-19 infection were less likely to have abnormal DLCO or desaturation with 6MWT at 1 year. As a secondary outcome, though not to statistical significance, those who initially received dupilumab trended toward reduced 1-year mortality compared to placebo. While we acknowledge the limitations in sample size and potential for survivor bias, it is important to note that we successfully achieved our primary objective in this study. This, combined with our preclinical data, provides evidence of a long-term benefit associated with IL-4Rα blockade during acute, severe COVID-19 infection.

Treatment with dupilumab for acute COVID-19 was also associated with a larger decline in eotaxin, IL-12p40, IP-10, and FLT3, and with an increase in the anti-inflammatory cytokine IL-1Ra. Previous studies have demonstrated persistent activation of T cells and innate immune cells for months after COVID-19 recovery, demonstrating prolonged immune activation in those with PCCs [33, 34]. Although this might suggest the need for prolonged anti-inflammatory therapeutics for prevention of PCCs, our study suggests that an acute type 2 immune blockade may deter a prolonged and destructive downstream immune cascade.

Dupilumab has been demonstrated to be a beneficial therapeutic in eosinophil-prominent subsets of both asthma and chronic obstructive pulmonary disease (COPD) [35, 36]. As those studies involved multiple treatments during the chronic stage of disease, what is striking here is that short-term treatment during acute COVID-19 appeared to have longer-term benefits.

Although no subjects originally enrolled in the trial presented with peripheral eosinophilia, when stratified by baseline lymphopenia, we found a significantly reduced mortality in the dupilumab group compared to placebo. While the mechanism of lymphopenia, whether it is due to viral-induced T cell apoptosis or increased tissue uptake of lymphocytes, is unknown, it has been associated with increased inflammation and worse outcomes in COVID-19 patients [32, 37]. This suggests that dupilumab may be most beneficial for those in the significant COVID-19–induced inflammatory state that leads to hospitalization.

Specific PFT abnormality differences related to oxygen diffusion seen in our study suggest downstream alterations at the alveolar-capillary interface, which could be explained by persistent endothelial activation and fibrosis [38]. We saw a nonsignificant trend toward reduced FEV_1_ in our dupilumab group compared to placebo. Recognizing the power limitations in our study, we would have expected FEV_1_ to be the predominant PFT difference seen in this study based on prior dupilumab studies in COPD and asthma [36, 39]. Our findings suggest that dupilumab was primarily able to inhibit a downstream vascular or perivascular fibrotic process induced by severe COVID-19, perhaps as a consequence of altered HA deposition [9].

We observed a larger reduction in peripheral eotaxin levels in our treatment group at 1 year, which was also seen in those who had normal PFTs compared to abnormal PFTs. As eotaxin is a chemokine that functions in the recruitment of eosinophils to promote eosinophil-induced inflammation [40], this demonstrated an association between diminished type 2 immune activity and normal pulmonary function after COVID-19. This trend was also visualized with IP-10, a chemokine downstream of interferon gamma (IFN-γ) [41], and TNF-α, a cytokine implicated in chronic inflammation and autoimmunity and also associated with PCCs [38, 42]. This further emphasizes dupilumab's anti-inflammatory effect and potential association with improved pulmonary function after COVID-19.

Wu et al observed that a third of nonventilated hospitalized patients for their COVID-19 illness exhibited reduced DLCO at 1 year, and Eberst et al reported 11% of intensive care unit (ICU) patients with reduced DLCO and 38% with abnormal 6MWT at 1 year [29, 30]. Comparison to our cohort (44% abnormal DLCO, 25% abnormal 6MWT in the cohort as a whole) suggests modestly higher rates of pulmonary dysfunction in our study population. This is consistent with our phase 2a findings, where we noted a higher disease severity in our subjects compared to other reports during the Delta wave [17]. Differences in our PFT findings from other studies may be due to the variation of PFT reference ranges used, PFT lab variability, SARS-CoV-2 variants involved (both observational studies included cohorts enrolled pre–Delta wave), preexisting comorbidities of subjects, or differences in COVID-19 management at the respective places and times. The 1-year mortality in our study was comparable to others as we found a 38% 1-year mortality in our placebo group compared to 30%–50% seen in other studies assessing those hospitalized for COVID-19 [43, 44].

Interestingly, we did not see a difference in reported symptoms, neurocognitive assessments, and CT findings between the 2 treatment groups as we might have expected, given the differences found in PFTs. Inconsistencies between subject perception of post-COVID-19 symptoms and measured PFTs have also been demonstrated in other post-COVID-19 studies [29, 38, 45]. As our study subjects did not have pre-COVID-19 assessments of these parameters for comparison, a possible explanation may be due to our inability to capture relative change in symptoms and PFTs from baseline measures. As we defined PFT abnormalities based on population-based norms, it is possible that patients suffered relative functional decline in their measures, which did not reach our predefined abnormality thresholds. That said, these subthreshold changes would likely require further study to determine their clinical implications. Lack of differences seen between CT scans may be reflective of vascular or microscopic parenchymal abnormalities that are not detectable on pulmonary imaging.

Limitations to this study include the small sample resulting from significant mortality in our placebo group, the subjects who declined follow-up visits, and the 2 subjects unable to complete full PFT assessment (either due to inability to follow maneuver directions or physical inability). While notably there were no statistically significant differences in initial viral load, comorbidities, demographics, therapies received, need for ICU, or vaccination status between the treatment groups of subjects who attended follow-up visits (Table 1), sample size limitations nevertheless constrain the conclusions we can draw from statistical analyses, particularly with regard to inclusion of model covariates. Additionally, although there were no statistically significant differences in clinical severity or demographics, either limiting functional capability to attend appointments or interest in appointments due to lack of clinical symptoms, between those who followed up compared to those who declined, we acknowledge the significant survivorship bias that exists in this study, which needs to be validated in a larger cohort.

Despite these limitations, the achievement of our primary endpoint, magnitude of separation between treatment groups and consistent trajectories of the PFT components observed between treatment groups, underscores the need for a larger, multicenter trial for further validation. We further acknowledge the need for a contemporary study when considering the change in population immunity with vaccination and reinfections from our phase 2a trial until now. Further supporting the need for additional study are the extensive preclinical human and mouse data demonstrating the significant contribution of a type 2 immune response to COVID-19–induced respiratory dysfunction, the findings from our phase 2a RCT, the favorable safety profile of dupilumab, and the therapeutic efficacy of dupilumab when used in other inflammatory pulmonary conditions. We conclude that dupilumab has promise as a therapeutic option in preventing post-COVID-19 oxygenation disturbance.

## Supplementary Data

Supplementary materials are available at *Open Forum Infectious Diseases* online. Consisting of data provided by the authors to benefit the reader, the posted materials are not copyedited and are the sole responsibility of the authors, so questions or comments should be addressed to the corresponding author.

## Supplementary Material

ofad630_Supplementary_Data

## Notes

***Acknowledgments.*** We thank the patients who consented to enroll in this study to help others with post-COVID-19 conditions; the staff at the University of Virginia (UVA) Post COVID clinic for their assistance with this follow-up study; and Amy Warren and Lori Elder for institutional review board (IRB) protocol preparation and Investigational New Drug (IND) preparation, submission, and maintenance. We also thank the Biorepository and Tissue Research Facility at UVA who collected, organized, and analyzed research samples.

***Patient consent*.** Approval for the original phase 2a study was obtained through the US Food and Drug Administration IND application in April 2021. Approval through the IRB at UVA was obtained for the original phase 2a trial in June 2021. The trial is registered at ClinicalTrials.gov (NCT04920916). This follow-up study was approved by the UVA IRB in June 2022. Consent forms for follow-up visits were IRB-approved. Each participant was provided a verbal explanation of the study purposes, study procedures, potential risks of the study, and their rights as research participants, which was suited to the participant's comprehension. Participants had the opportunity to carefully review the written consent form, discuss with family or surrogates, and ask questions prior to signing.

***Financial support*.** This work was supported by the Virginia Biosciences Health Research Corporation; PBM C19 Research, LLC of PBM Capital; the Henske Family Foundation; and the National Institutes of Health (grant numbers R01 AI124214 to W. A. P., T32 DK072922 and TL1 DK132771 to J. H., and UL1TR003015 and KL2TR003016 to J. M. S. and J. Z. M.). J. H. is an Integrated Translational Health Research Institute of Virginia (iTHRIV) Scholar. The iTHRIV Scholars Program is supported in part by the National Center for Advancing Translational Sciences of the National Institutes of Health under award numbers UL1TR003015 and KL2TR003016. J. E. A. was funded by the Medical Research Council UK (MR/V011235/1) and the Wellcome Trust (106898/A/15/Z).
