# Supplementary material for: Pulmonary Function and Survival 1 Year After Dupilumab Treatment of Acute Moderate to Severe Coronavirus Disease 2019: A Follow-up Study From a Phase 2a Trial
Source: Open Forum Infect Dis. 2024 Jan 3;11(2):ofad630. doi: 10.1093/ofid/ofad630 (PMC10834240; doi:10.1093/ofid/ofad630)
Supplement: ofad630_Supplementary_Data [file ofad630_supplementary_data.docx]

**SUPPLEMENTAL DATA**

**Pulmonary function and survival one year after dupilumab treatment of acute moderate to severe COVID-19: A follow up study from a Phase IIa trial**

Jennifer Hendrick^1^, Jennie Z. Ma^2^, Heather M. Haughey^3^, Rachael Coleman^1^, Uma Nayak^4^, Alexandra Kadl^3,5^, Jeffrey M. Sturek^3^, Patrick Jackson^1^, Mary K. Young^1^, Judith E. Allen^6^, William A. Petri, Jr^1,7,8*^

^1^Division of Infectious Diseases and International Health, Department of Medicine, University of Virginia Health System, Charlottesville, VA, USA;

^2^Department of Public Health Sciences, University of Virginia School of Medicine, Charlottesville, Virginia, USA;

^3^Division of Pulmonary and Critical Care Medicine, Department of Medicine, University of Virginia Health System, Charlottesville, VA, USA;

^4^Center for Public Health Genomics and Department of Public Health Sciences, University of Virginia School of Medicine, Charlottesville, VA, USA;

^5^Department of Pharmacology, University of Virginia School of Medicine, Charlottesville, VA, USA;

^6^Lydia Becker Institute of Immunology and Inflammation, School of Biological Sciences, University of Manchester, Manchester Academic Health Sciences Centre, Manchester, United Kingdom;

^7^Department of Microbiology, Immunology and Cancer Biology, University of Virginia School of Medicine, Charlottesville, VA, USA;

^8^Department of Pathology, University of Virginia Health System, Charlottesville, VA, USA

*Corresponding author: William A. Petri Jr. University of Virginia, 345 Crispell Drive, Charlottesville Virginia 22908-1340, USA. Email: wap3g@uvahealth.org; Phone: 434.924.5621. ORCID ID 0000-0002-7268-1218


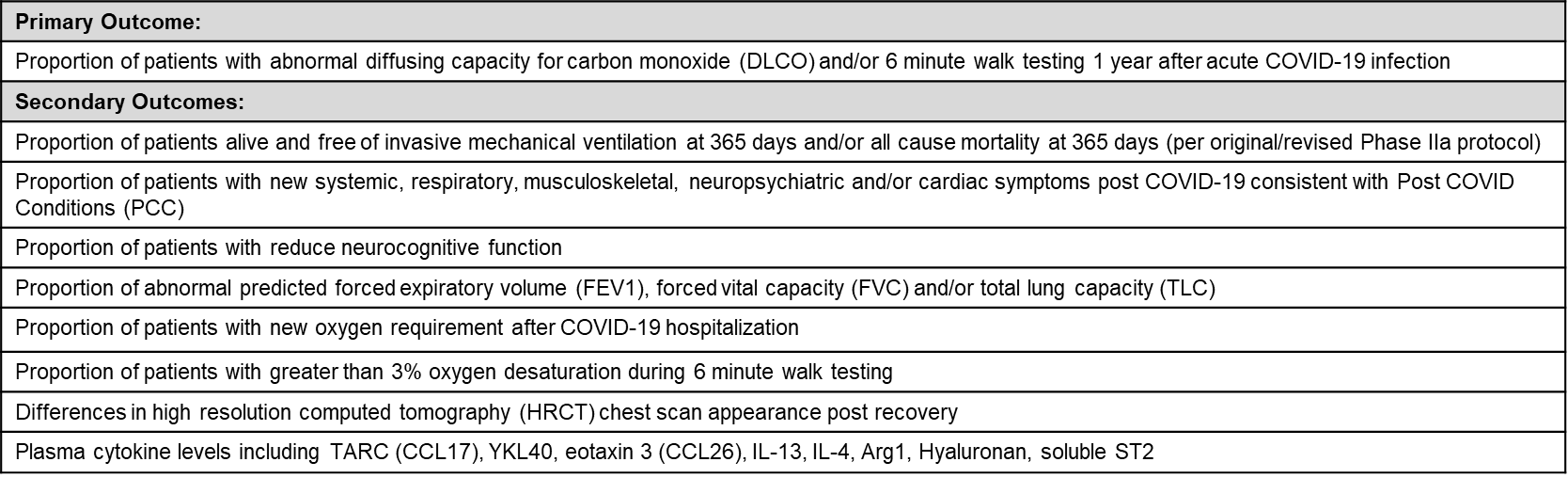


Table S1: Primary and secondary outcomes. All data was collected at 1 year follow-up appointments and/or phone calls at 1 year post Phase IIa enrollment.

Table S2: Neurocognitive tests administered at 1 year follow up visits.


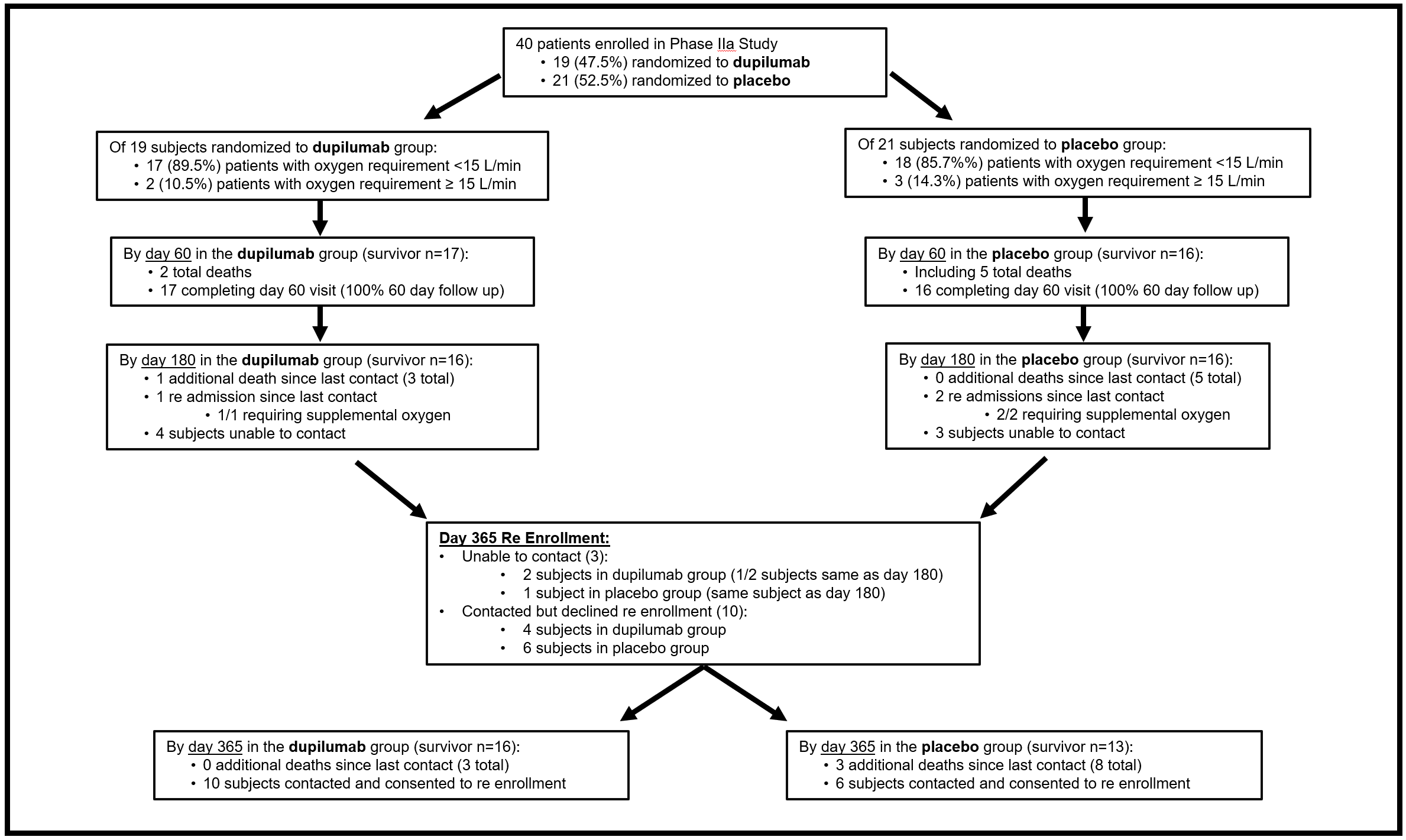


Fig S1: Study timeline and re-enrollment. Survival data was extracted from the electronic medical record (EMR) in those subjects that were unable to be contacted by telephone.


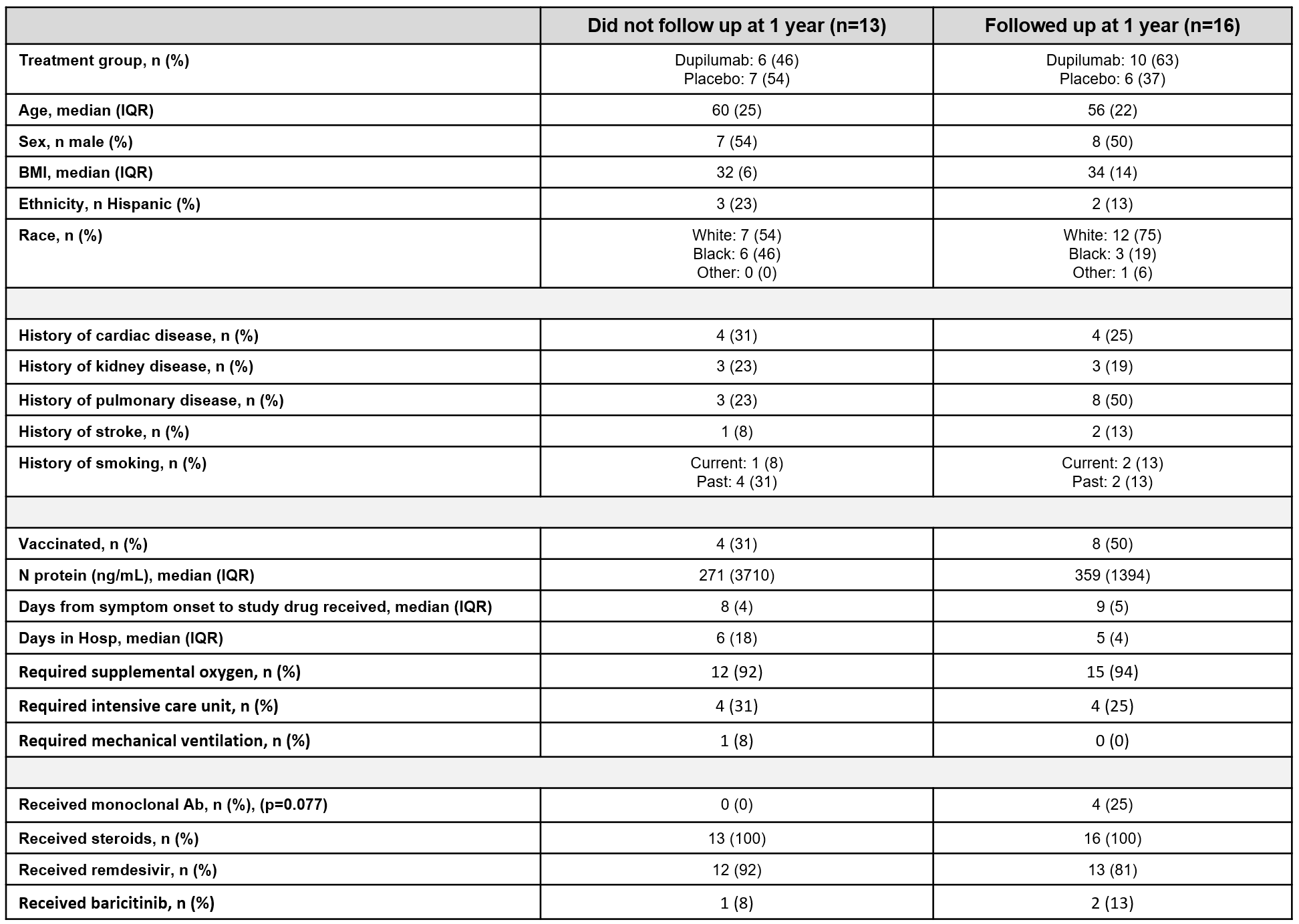


Table S3: Demographics, co morbidities, clinical characteristics and medications received while during COVID-19 admission between those who consented to follow up visit versus those who were alive and declined follow up visit at 1 year post enrollment.


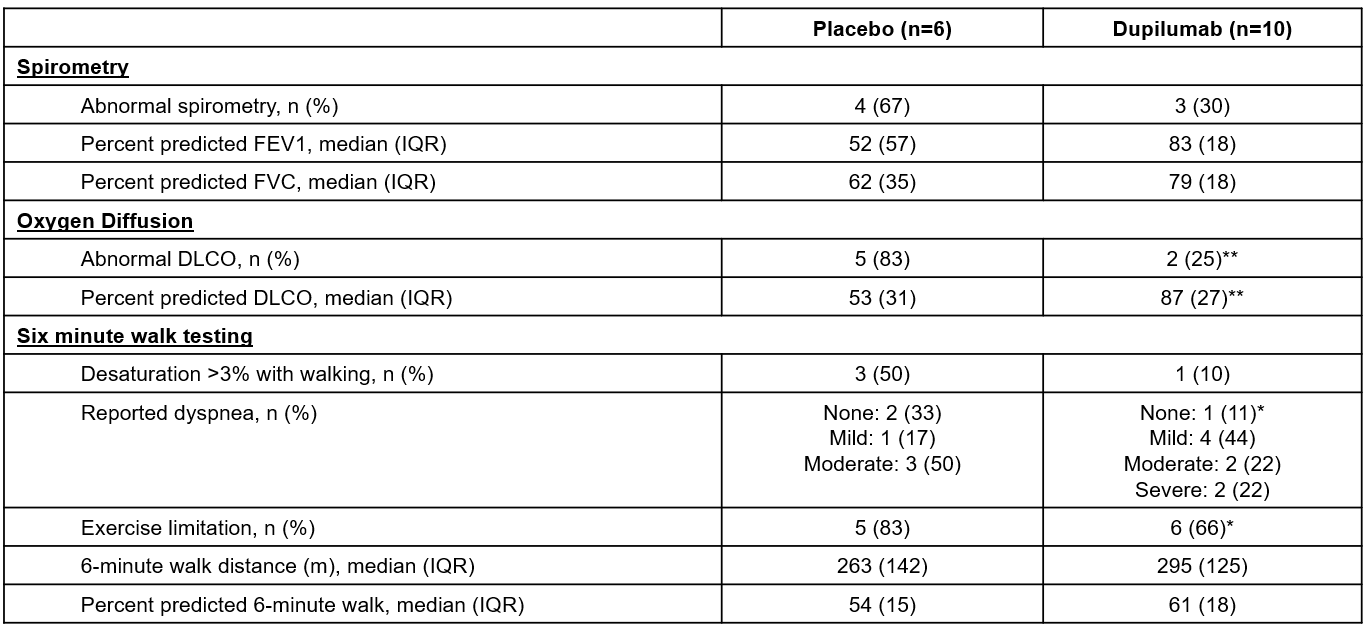


Table S4: Pulmonary function testing measurements by treatment group at 1 year follow up. Spirometry and oxygen diffusion data is displayed as a continuous measure indicating percent of predicted compared to Global Lung Function Initiative (GLI) predicted values based on age, sex, height and ethnicity. This data is also displayed as a binary measure (% abnormal) which depicts the interpretation of measurements. Six-minute walk testing is displayed as continuous measures for actual distance walked and percent of predicted as determined by subject age, sex, height and weight. It is also displayed as a binary variable indicating percent of subjects who desaturated, who reported dyspnea and who displayed exercise limitation during their six-minute walk. * indicates 1 missing value ** indicates 2 missing values.


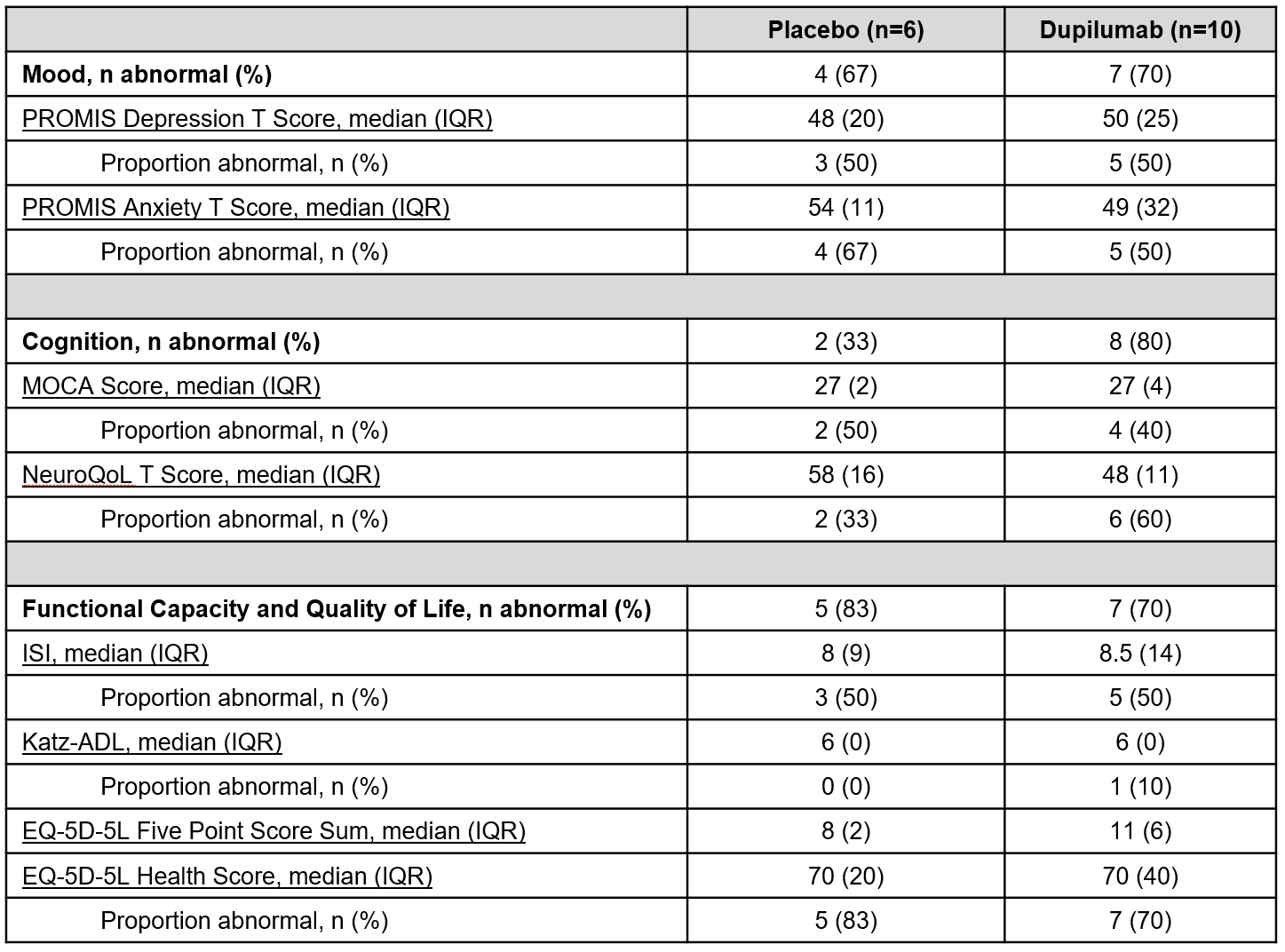


Table S5: Neurocognitive testing results at 1 year follow up by treatment group. Each test is split into three types of measurement categories: mood, cognition and functional capacity. Measurements are displayed as a score and interpretation (% abnormal) of those scores for that treatment group. The EQ-5D-5L five-point score raw numbers are displayed as a median of the sum of all 5 scores and as a median of reported health score (on scale from 0-100) for each treatment group.


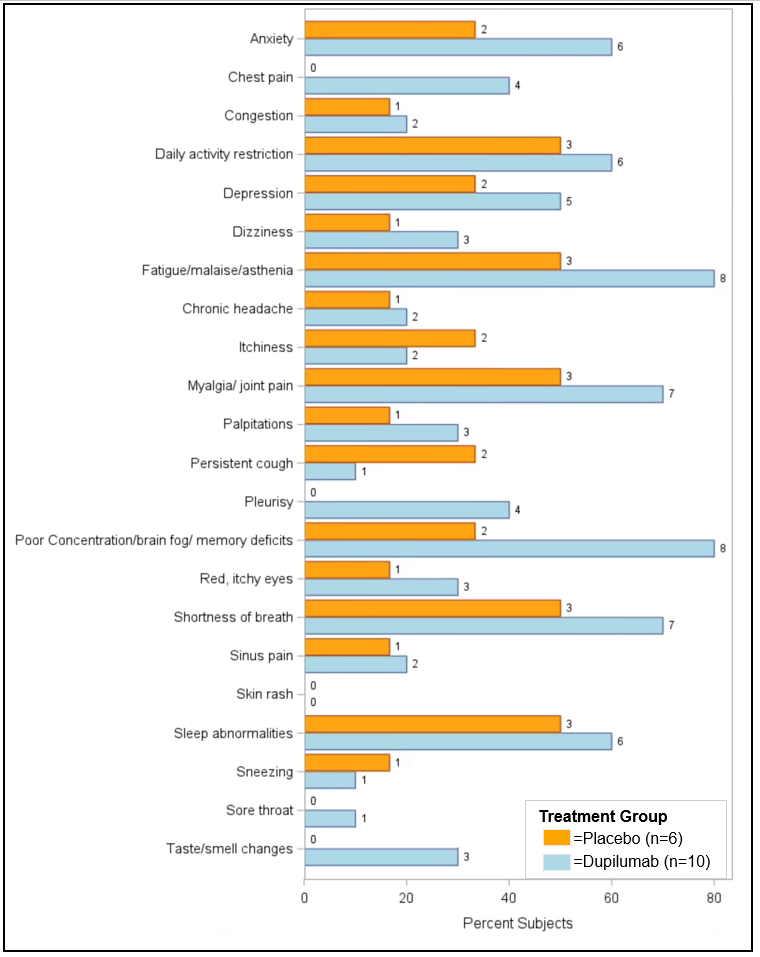


Fig S2: Symptoms reported at 1 year follow up by treatment group. The horizontal axis indicates the percent of patients who followed up and reported that symptom. The number at the end of each bar represents raw count out of the total for that treatment group. The orange boxes depict the patients randomized to placebo during initial COVID-19 admission and blue box depicts the subjects randomized to dupilumab during initial COVID-19 admission.


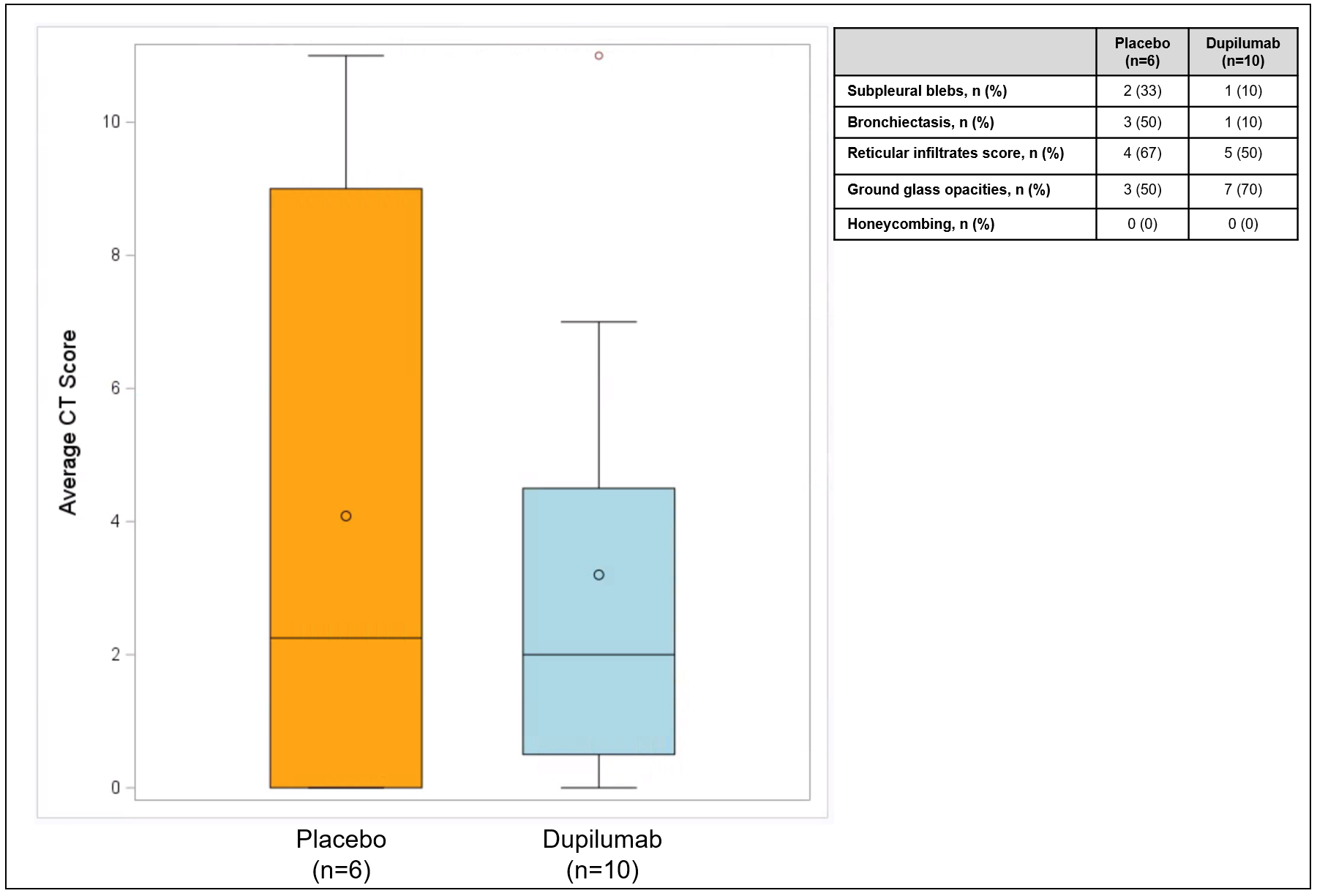


Fig S3: Computer tomography (CT) scores and abnormalities seen by treatment group. CT scores are depicted in the box and whisker plot on the left and were generated as an average of scores from two independent pulmonologists. Scores were generated by totaling scores based on parenchymal findings from three areas of the lung. The orange box depicts the patients randomized to placebo and the blue box depicts the subjects randomized to dupilumab during initial COVID-19 admission. Solid horizontal line within box is representative of median value and open circle within box is representative of mean value. The table on the right shows number (n) and percentage (%) of subjects who displayed those parenchymal abnormalities at any lung level.

Table S6: Table showing median (interquartile range) (IQR) serum cytokine, chemokine or growth factor differences (column on far left) between day 365 and day 0 by treatment group (left side) and by PFT status (right side). P values are generated from Wilcoxon test. PFT= pulmonary function testing. Further, the false discovery rate (FDR) analysis for multiple comparison showed that none of cytokine, chemokine and growth factor analyses remained significantly different (all FDR-adjusted p>0.39), whether by treatment groups or by PFT statuses.


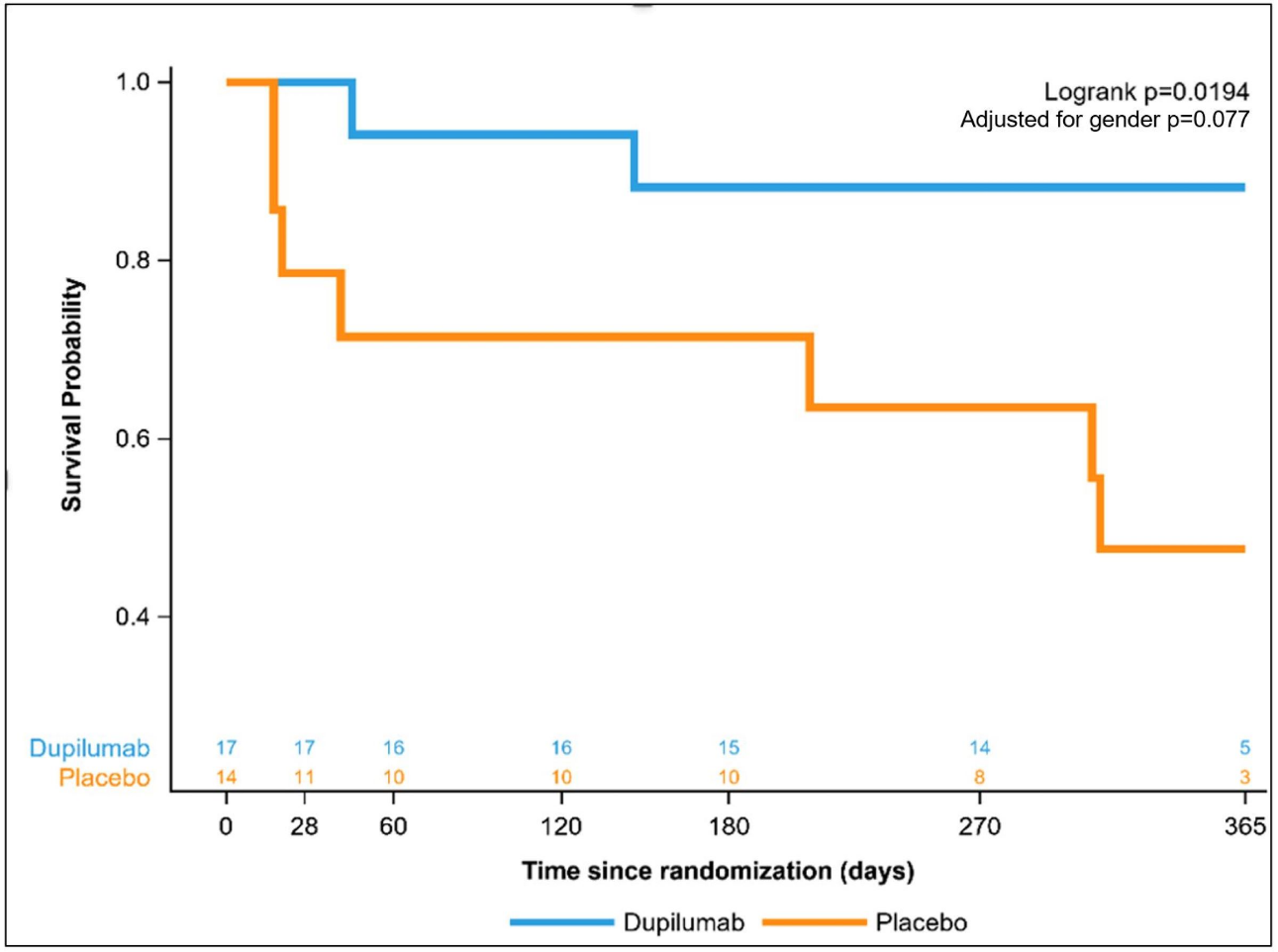


Fig S4: Kaplan Meier curve depicting 1-year mortality between the two treatment groups stratified by lymphopenia (defined as a lymphocyte count less than 1,000 cells/µL). Dupilumab group is represented by blue line. Placebo group is represented by the orange line. Adjusted p value indicative of adjustment for sex in the Cox regression.


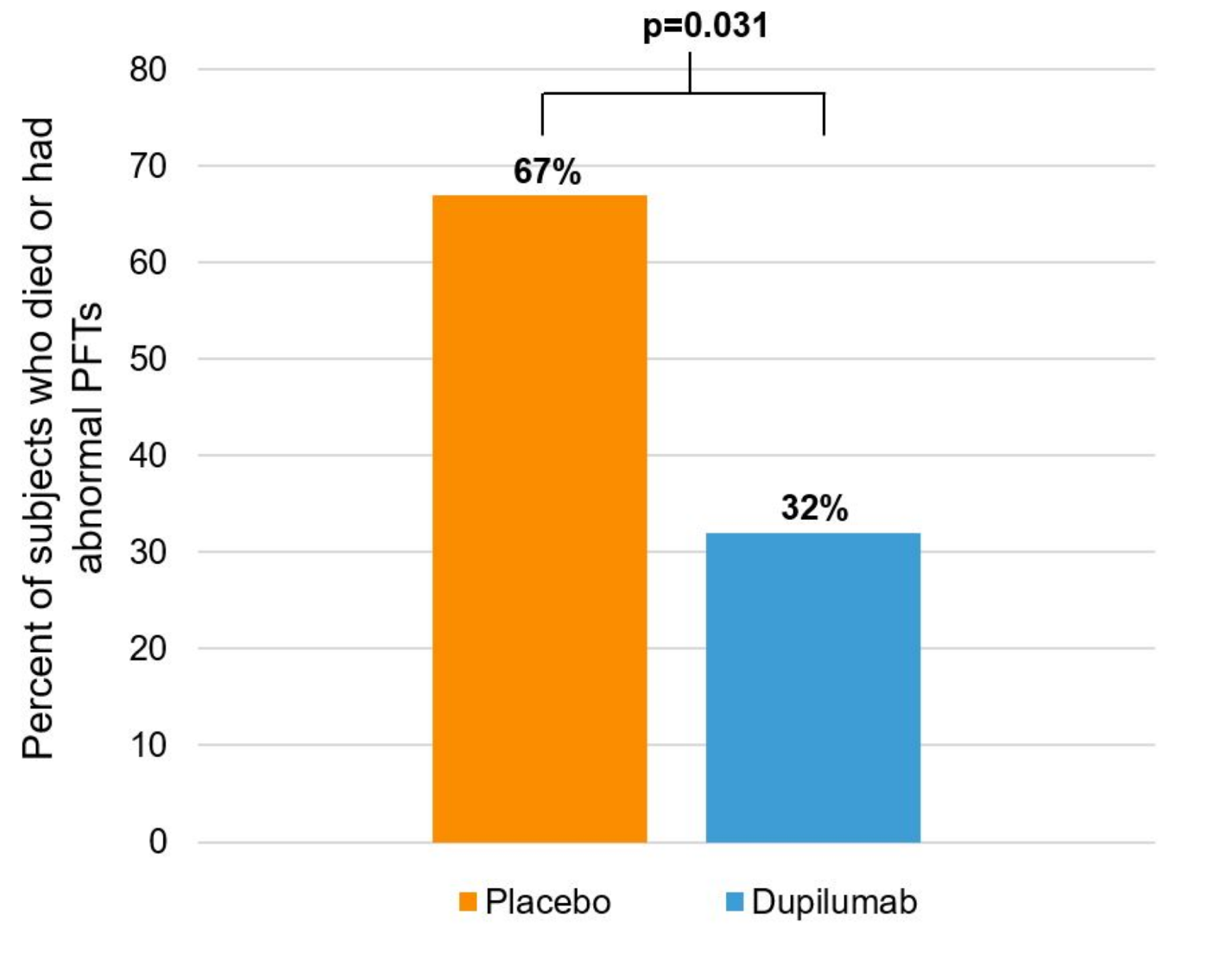


Fig S5: Bar graph illustrating the percent of patients who died or had abnormal pulmonary function testing (defined by abnormal DLCO or 6MWT) by treatment group at 1-year follow up. The orange box depicts the patients randomized to placebo during initial COVID-19 admission and blue box depicts the subjects randomized to dupilumab during initial COVID-19 admission. Actual percent displayed at the top of each bar for corresponding treatment group.
